# Combining socioeconomic and biophysical data to identify people-centric restoration opportunities

**DOI:** 10.1038/s44185-023-00012-8

**Published:** 2023-03-01

**Authors:** Pooja Choksi, Arun Agrawal, Ivan Bialy, Rohini Chaturvedi, Kyle Frankel Davis, Shalini Dhyani, Forrest Fleischman, Jonas Lechner, Harini Nagendra, Veena Srininvasan, Ruth DeFries

**Affiliations:** 1 Department of Ecology, Evolution, and Environmental Biology, Columbia University, New York, New York USA; 2 School of Environment and Sustainability, University of Michigan, Ann Arbor, MI USA; 3 University of Edinburgh, Edinburgh, Scotland UK; 4 Global EverGreening Alliance, Victoria, Australia; 5 Department of Geography and Spatial Sciences, University of Delaware, Newark, DE USA; 6 Department of Plant and Soil Sciences, University of Delaware, Newark, DE USA; 7 CSIR National Environmental Engineering Research Institute, Nagpur, Maharashtra India; 8 Department of Forest Resources, University of Minnesota, St. Paul, MN USA; 9 Unique landuse GmbH, Freiburg, Germany; 10 School of Development, Azim Premji University, Bengaluru, Karnataka India; 11 Ashoka Trust for Research in Ecology and the Environment, Bangalore, Karnataka India

**Keywords:** Restoration ecology, Environmental social sciences

## Abstract

Designing restoration projects requires integrating socio-economic and cultural needs of local stakeholders for enduring and just outcomes. Using India as a case study, we demonstrate a people-centric approach to help policymakers translate global restoration prioritization studies for application to a country-specific context and to identify different socio-environmental conditions restoration programs could consider when siting projects. Focusing, in particular, on poverty quantified by living standards and land tenure, we find that of the 579 districts considered here, 116 of the poorest districts have high biophysical restoration potential (upper 50th percentile of both factors). In most districts, the predominant land tenure is private, indicating an opportunity to focus on agri-pastoral restoration over carbon and forest-based restoration projects.

Ecological restoration is a crucial nature-based solution for carbon sequestration and biodiversity conservation^1^. To fulfill targets of the Nationally Determined Contributions, the Bonn Challenge^2^ and land degradation neutrality^3^, research has identified areas of high value to restoration across the world based on biophysical characteristics^4–6^. While global restoration studies and prospecting tools enable private and public entities to decide where to focus restoration efforts for maximum biodiversity and carbon sequestration value, they leave people off the map. Designing and siting successful restoration projects requires consideration and integration of socio-economic needs and cultural characteristics of local stakeholders. Although there is an increasing recognition that local people need to be engaged and their interests need recognition in the design and implementation of restoration projects^7,8^, there are few examples of systematic consideration of people’s livelihoods and interests in restoration at large spatial scales^9^. Coarse socio-economic datasets cannot replace local consultations and needs assessments to ensure restoration projects provide benefits to local people. However, these data can be used as preliminary filters for different restoration methods. Here, we propose an explicit consideration of people’s socio-economic needs through the combination of biophysical and socio-economic factors to identify people-centric restoration opportunities. We also assess the *de jure* land tenure system to identify which types of land could be targeted for more tenure-responsive, long-lasting and socially just outcomes^10^.

We use India as a case study as it has a high biophysical restoration potential^5,6^ and one of the largest restoration targets of 26 million hectares by 2030^11^. A large proportion (64%) of India’s population is rural and relies on local ecosystems for livelihoods through small-scale agriculture and common pool resources, making a people-centric lens to restoration design and implementation necessary. India’s focus on socio-economic development through programs such as the Aspirational Districts Programme^12^, emphasizes the need for the environmental agenda to align with the development agenda. For this analysis, we thus consider the living standards component of the multidimensional poverty as our socio-economic metric at the district level (*N* = 579 districts) to reflect dependence on natural resources. We choose this metric because people more dependent on natural resources for their subsistence and livelihoods are more likely to (a) be vulnerable to decisions made regarding land uses and (b) benefit from improved availability of natural resources in the short term. We compare this metric with the biophysical restoration potential (as quantified in Strassburg et al. 2020^6^) to identify different socio-environmental conditions restoration programs must consider in order to balance environmental and social goals. Furthermore, we classify *de jure* land tenure regimes by aggregating village-level census data^13^ to identify prevalent land tenures. Land tenure is important for understanding who may have the authority to change land use. Although the biophysical restoration potential considered in this study refers to restoration without human disturbance^6^, we argue that such restoration is challenging and socially unjust in a country with high human population densities. Therefore, we define restoration as any activity which restores ecological functionality to degraded landscapes^2^, ranging from alternative agricultural and pastoral practices to natural ecosystem restoration.

We find that approximately 29% of districts (*N* = 166) with high biophysical potential are also above average poverty levels in India (above 50th percentile for biophysical potential and poverty of 579 districts; Figs. 1 and 2 quadrant 1). Similarly, 30% (*N* = 168) of districts have both below average biophysical potential and below average poverty (below 50th percentile for biophysical potential and poverty; Fig. 2, quadrant 3). This overlap indicates the potential and need to pursue restoration in a manner that addresses both ecological and social goals.

**Fig. 1:**
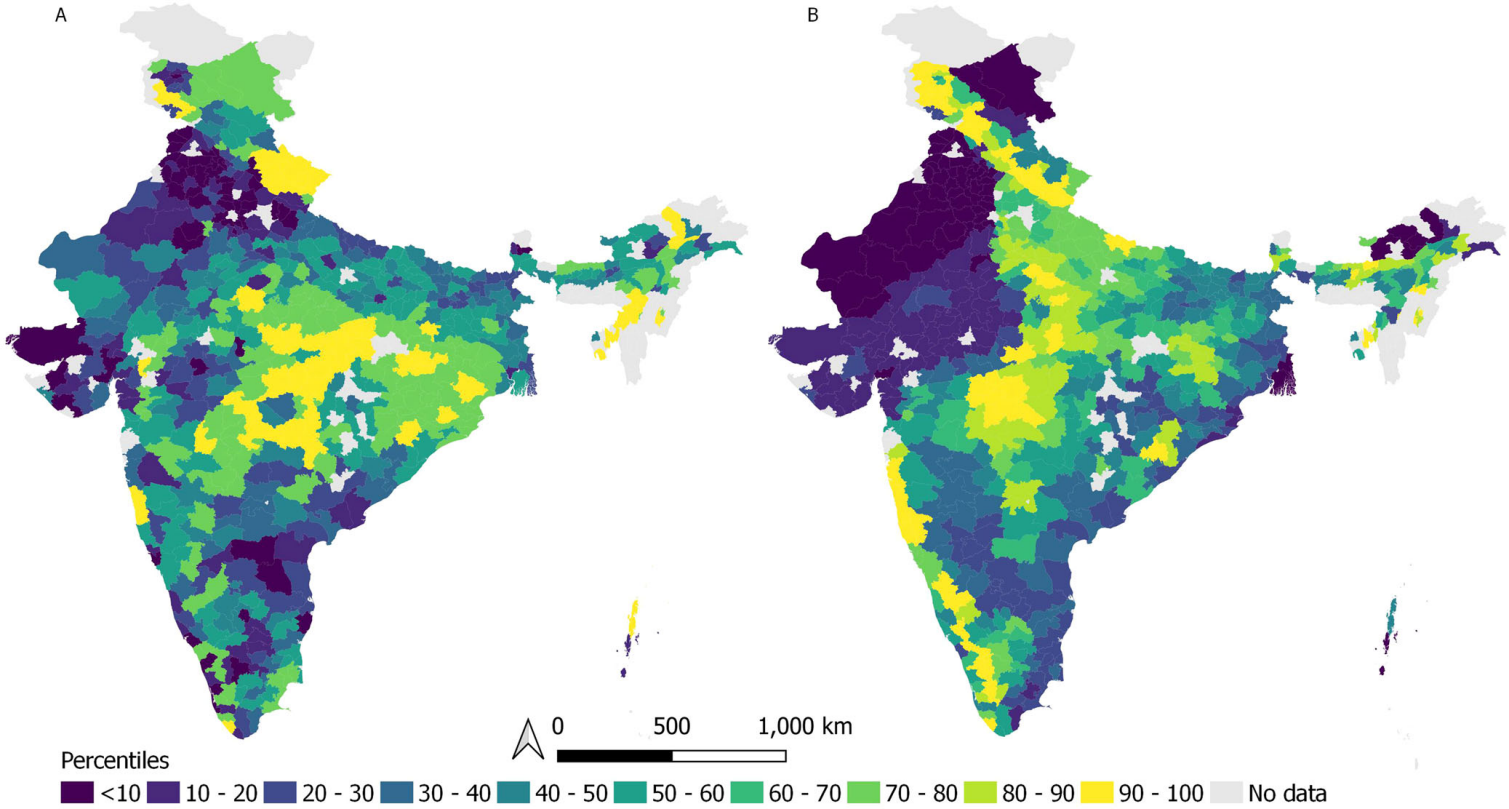
Map of India displaying districts mapped according to variables considered in this study. **A** Living standards component of the Multidimensional Poverty Index and **B** Biophysical restoration potential (quantified by Strassburg et al. 2020^6^). The colors represent the percentile range to which the districts belong.

**Fig. 2:**
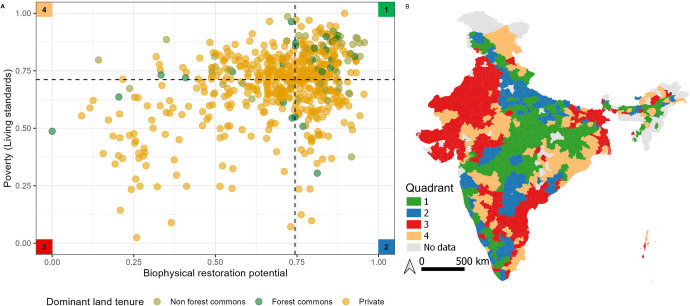
A comparison of each district’s biophysical potential and poverty level. **A** Districts plotted in reference to biophysical restoration potential and poverty measured by the living standards component of multidimensional poverty. Each district is presented as a circle. Colors represent the dominant land tenure in the district. Vertical and horizontal dashed lines represent the 50th percentile according to biophysical restoration potential and poverty. The numbers in the corner of each quadrant correspond to districts of the same color in **B**.

In the majority of the 579 districts considered in this study, private land is the predominant land tenure, followed by non-forest commons, then forest commons (Fig. 3). Although recent restoration efforts have overwhelmingly focused on afforestation^14,15^, recent evidence indicates a larger climate change mitigation potential in alternative agricultural systems, such as agroforestry and trees outside forests (ToF), than in areas which are likely to be managed as closed-canopy forests^16^. Furthermore, the disproportionate focus on carbon-centric forest-based projects has led to underrepresentation of projects aimed at reducing emissions of other greenhouse gases (GHGs) such as methane with enormous mitigation potential^14^. Traditional agroforestry practices and ToF (e.g., live fences, silvi-pastures, horti-pastoral systems) are common in India^17^ and could lower other GHG emissions. While it may be simpler to facilitate agroforestry among individual land holders with clear land titles; restoring degraded common lands may facilitate broader benefits, particularly among the poorest people who often don’t own land or have a strong culture of common ownership (e.g., pastoralist communities in Gujarat and Rajasthan). However, restoration of the commons can be complex when the source of degradation (e.g., an invasive species), becomes a source of livelihood for a section of the local community^18^.

**Fig. 3:**
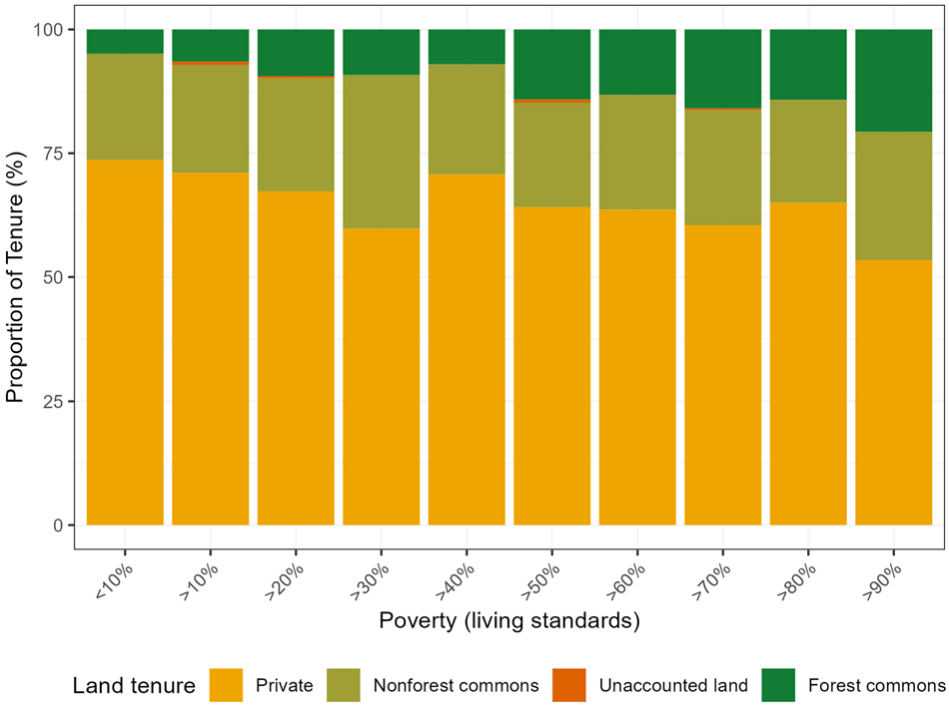
The proportion of each land tenure in the 579 districts belonging to the ten percentiles in ascending order. Districts above 90th percentile are poorer than districts under the 10th percentile.

By analyzing biophysical, socio-economic and land tenure data together, policy makers can devise restoration programs more holistically. For example, ten of the fourteen poorest districts that have very high biophysical restoration potential (above 90th percentile in both restoration potential and poverty), have a predominance (>50%) of non-forest (*N* = 8) and forest commons (*N* = 2). In districts above the 80th percentile in terms of both restoration potential and poverty, approximately 40% had a predominant land tenure of forest (*N* = 9) and non-forest commons (*N* = 9, total = 45 districts). It may be tempting to situate reforestation and afforestation projects, which are based mainly on plantation models^15^, in poorer districts with high value for restoration. However, emerging evidence shows that afforestation projects do not always increase forest cover^19^, sometimes reduce pastoralist access to grazing lands^20^, and do not contribute much to the local communities’ needs for firewood and fodder^19^. We argue that in districts with high biophysical restoration potential and high poverty, it could be more effective to (a) encourage traditional agroforestry practices, (b) leverage economic policies and schemes designed to raise living standards^21^, (c) use alternative restoration practices, such as invasive species management in districts with a high proportion of common land and (d) allow for greater community rights to manage the commons^22^. For example, approximately 30% of the districts above the 80% percentile of both restoration potential and poverty are in Madhya Pradesh. Managing an invasive species, *Lantana camara* in forest and non-forest commons in that state increased the local communities’ access to firewood and fodder^15^. Moreover, recent evidence from some of these districts shows that switching to alternative energy sources for cooking and use of durable housing materials raised living standards, as well as provided a safer cooking fuel option and contributed to forest regeneration near villages^21^.

Similar evidence of forest regeneration with the adoption of biogas digesters in a district with high poverty but low biophysical restoration potential, such as Chikkaballapur in Karnataka, emphasizes the potential of human well-being policies to have positive ecological outcomes^23^. In districts with high biophysical restoration potential and low poverty, including Malappuram and Thrissur in Kerala, agroforestry and cash crop plantations, along with other livelihood alternatives, have played a role in alleviating poverty and increasing food security^24^. These traditional agroforestry systems and private home gardens could continue to be supported and incentivized. Furthermore, novel tools such as Diversity for Restoration (D4R) help people select appropriate species for planting based on the outcomes they are interested in, such as erosion control^25^. In regions with low poverty and low biophysical potential (both factors below 50th percentile), such as districts in Rajasthan and Gujarat, the predominant land tenure is private. These districts could be targeted for irrigation management to increase drought resistance and agri-pastoral projects which could simultaneously contribute to reductions in methane emissions^14,17^. With a considerable area of non-forest commons (>33.33% land tenure), pasture and open natural ecosystems (ONEs) restoration could also be beneficial to the numerous indigenous pastoralist communities in these states^26,27^. Moreover, ONEs would not necessarily store more carbon if afforested^28^. Thus, preserving these non-forest ecosystems will not only benefit pastoralists but also conserve unique non-forest ecosystem biodiversity^27,28^. The interventions suggested in the four different socio-environmental conditions were not designed in the context of the relationship between biophysical restoration potential and poverty. Therefore, it is critical to understand the applicability of these interventions in the context of these different conditions, and the cost-effectiveness of these interventions to successfully scale them.

Our analysis has some limitations. First, the district administrative unit is a convenient spatial scale to plan interventions and programs. But we recognize that households are not socio-economically uniform and thus, restoration programs will not have uniform effects in a district. As an example, agroforestry programs can have very different food security outcomes for people who own land and those who do not. Second, the analysis carries inherent uncertainties found in the data sources.

This study attempts to demonstrate a people-centric approach to translating global biophysical restoration potential studies for application to a country-specific context, rather than prescribing restoration priorities. Based on a country’s development and environmental agenda, the variables used to determine the different socio-environmental conditions may be different. An analysis of this nature can help policy makers and an emerging diversity of actors in the field of ecological restoration broadly filter restoration methods best suited for different socio-environmental conditions.

## Methods

### Data sources and preparation

#### Land uses and de *jure* land tenure regimes

We aggregated the most recent publicly available census data (2011)^13^ at the village level to the district level to quantify the *de jure* land tenure regimes that include private land, common non-forest land and forest land. For this study, we consider 579 districts for which we had a complete dataset, including the data on poverty and biophysical restoration potential. We categorized the land use data available at the census village level into the following *de jure* land tenures:

**Table:** 

Land tenure regime	Land use categories from Census 2011 land records
Private land	1. Net sown area 2. Current fallow land 3. Fallow lands other than current fallows
Common non-forest land	1. Culturable wastelands (grasslands) 2. Area under non-agricultural use 3. Barren or uncultivable land 4. Permanent pastures or grazing lands 5. Land under miscellaneous tree crops (orchards)
Common forest land	Forest

In order to only include inhabited census villages, we removed census villages with zero as total population and those explicitly labeled ‘uninhabited’ in the village name. Further, we included only non-state-owned land by filtering out the following categories of census villages:

**Table:** 

Type of state-owned land	Terms used in the census village name
Army owned land or firing range	*firing range*
Forest	*reserve, beat, block, forest, camp, range, gate, K.M*.

In order to report the total hectares of specific land uses and to calculate the proportion of *de jure* land tenures, we treated any inconsistencies in the original census land use data in the following manner:

**Table:** 

Inconsistency in the land use records	Description of the inconsistency	Potential reason for inconsistency	Treatment of inconsistency
No land use records	All land use columns show zero hectares but Total area in hectares has a positive value	The census enumerators did not reach these villages.	These villages appear as ‘*No data’*
Total areas in hectares reported not equal to total of all land uses	Column from Census 2011 records not equal to actual total hectares of all land uses. There are two possibilities: a: Total area in hectares > total of all land uses or b. Total area in hectares < total of all land uses	Error in addition of land uses by census enumerator or land use is currently disputed.	1. For our analysis, we considered the total of all land uses to calculate the proportion of land tenure for a village. 2. We created a variable ‘*Unaccounted land*’ = Total area in hectares- Total of all land uses
Total area in hectares is reported as zero but land use records exist	All land use columns have a positive value in hectares but Total area in hectares is zero	Error in addition of land uses by census enumerator.	For our analysis, we considered the total of all land uses to calculate the proportion of land tenure for a village.

#### Living standards component of the multidimensional poverty index

Our study used one dimension (living standards) of the three dimensions of the multidimensional poverty index (living standards, health and education)^29^. We chose to only look at the percent contribution of living standards to poverty in a district because education and health services are provided largely by the government and may not necessarily reflect poverty due to the lack of viable livelihood options. For 579 districts, the percent contribution of living standards to multidimensional poverty ranged from 18.2% to 56.7%. We scaled this percentage from 0 to 1 to ensure that we could make a fair comparison with the biophysical potential for restoration taken from Strassburg et al. 2020^6^. We split the districts into 10 percentiles based on their value, with values closer to zero indicating higher living standards and 1 denoting lower living standards or higher levels of poverty (Fig. 1a).

#### Biophysical potential for restoration

We used the spatial data from Fig. 1e from Strassburg et al. 2020, which considers the ecological restoration potential of countries around the world based on the biodiversity conservation and climate change mitigation potential that a location holds while considering the cost of land. In R computing software, using the packages *raster*^30^ and *rgdal*^31^, we clipped the map of the restoration potential of the districts in India to compute the mean biophysical restoration potential of a district. The values of the original dataset ranged from 1 to 20, denoting 5% increments in restoration potential. We rescaled the values from 0 to 1 to make a fair comparison with the living standards component of the multidimensional poverty index. We split the 579 districts into 10 percentiles for presentation (Fig. 1b).

All maps in this study were created using QGIS version 3.16.8^32^.

### Reporting summary

Further information on research design is available in the Nature Research Reporting Summary linked to this article.

### Supplementary information

Reporting Summary

## Data Availability

All the data used in this analysis is publicly available and available from the authors of Strassburg et al. 2020.

## Code availability

The R code used to create figures and data used for mapping is available on GitHub.
